# Elucidating the role of missense SNP of protein kinase C epsilon in HCV-induced hepatocellular carcinoma

**DOI:** 10.1186/s12885-023-10618-7

**Published:** 2023-02-13

**Authors:** Areeba Rehman, Maria Shabbir, Yasmin Badshah, Khushbukhat Khan, Janeen H. Trembley, Naeem Mahmood Ashraf, Tayyaba Afsar, Ali Almajwal, Nawaf W. Alruwaili, Ali Alshamari, Tariq Nahar Alanezi, Suhail Razak

**Affiliations:** 1 Atta-ur-Rahman School of Applied Biosciences (ASAB), National University of Sciences and Technology (NUST), Islamabad, Pakistan; 2 School of Biochemistry & Biotechnology, University of the Punjab, Lahore, Pakistan; 3 Minneapolis VA Health Care System Research Service, Minneapolis, MN USA; 4 Department of Laboratory Medicine and Pathology, University of Minnesota, Minneapolis, MN USA; 5 Masonic Cancer Center, University of Minnesota, Minneapolis, MN USA; 6 Department of Community Health Sciences, College of Applied Medical Sciences, King Saud University, Riyadh, Saudi Arabia; 7 College of Medicine, King Saud University, 11481 Riyadh, Saudi Arabia

**Keywords:** Protein kinase C (PKC), Hepatocellular carcinoma (HCC), Single nucleotide polymorphism (SNP), Hepatitis C virus (HCV)

## Abstract

**Background:**

The protein kinase C (PKC) family of serine/threonine kinases contains more than ten isozymes that are involved in multiple signaling pathways, including cell cycle regulation and carcinogenesis. The PKCε isozyme is an oncogene known to be upregulated in various signaling pathways involved in hepatitis C virus (HCV)-induced hepatocellular carcinoma (HCC). However, there is no known association of missense SNPs in PKCε with this disease, which can be a potential biomarker for early diagnosis and treatment. This research reveals a novel missense SNP in PKCε that is associated with HCV-induced HCC in the Pakistani population.

**Methods:**

The PKCε SNP with amino acid substitution of E14K was chosen for wet lab analysis. Tetra ARMS-PCR was employed for the identification of high-risk SNP in PKCε of HCV-induced HCC patients. Liver function testing was also performed for comparison between the liver condition of the HCC patient and control group, and the viral load of HCC patient samples was evaluated to determine any alteration in the viral infectivity between different genotypes of the selected high-risk PKCε variant SNP.

**Results:**

Frequency distribution of the homozygous GG genotype was found to be highest among HCV-induced HCC patients and was also found to be significantly associated with disease development and progression. The *p* values of comparative data obtained for the other two genotypes, heterozygous AG and homozygous AA, of the SNP also showed the significance of the data for these alleles. Still, their odds ratio and relative risk analysis did not indicate their association with HCV-induced HCC.

**Conclusion:**

The distribution of a genotype GG of PKCε has been found in HCV- induced HCC patients. Therefore, these PKCε SNP have the potential to be biomarkers for HCV-induced HCC. Further investigation using a larger sample size would provide additional insight into these initial data and open a new avenue for a better prognosis of this disease.

## Background

Protein kinase C (PKC) is a large family of serine-threonine kinases which are expressed in various types of cells. More than ten types of PKC isozymes have been discovered so far which have been categorized into three sub-classes, the classical cPKCs (α, β1, β2, γ), novel nPKCs (δ, ε, η), and atypical aPKCs (λ, ζ) [1]. PKCε is a calcium independent and phorbol ester /Diacyl glycerol (DAG) dependent isoform in the novel PKC subclass. It is activated and compartmentalized to specific cellular sites depending on the secondary messengers and the adaptor proteins activated in response to the extracellular stimuli [2, 3]. Various receptors like tyrosine kinase receptors, tyrosine kinase-coupled receptors and G-protein-coupled receptors can lead to PKCε induction [4], which in turn is involved in various physiological processes such as immune system activation, inflammatory responses, exocrine, endocrine and nervous system stimulation [5, 6]. PKCε has been positively associated with several kinds of cancers and plays major roles in several tumorigenic pathways [7–11]. Various studies have shown that PKCε directly phosphorylates AKT at S473 [12] and SMAD3 at S213 [13], activating their respective pathways resulting in uncontrolled cell survival and proliferation. Evidence indicates the role of PKCε in modulating the expression of AFP, which has significance as a diagnostic and follow-up marker of HCC [14]. PKCε has also shown to facilitate hepatic steatosis and impairment of hepatic insulin signaling, thus, promoting insulin resistance which is a major factor in liver disease and development of HCC in patients with chronic HCV infection [15–17]. Besides that, PKCε activates ATF2 signaling which is involved in CAP2 expression that is induced in conditions of ER stress and promotes epithelial to mesenchymal transformation (EMT), an important hallmark of cancer, via Rac1 and ERK in HCC [18].

Most of the data available so far have only indicated the carcinogenic role of PKCε at the functional level. However, genotype association studies for other PKC isoforms with cancer susceptibility have been conducted, indicating the pathogenic effect of certain SNPs [19, 20]. Recently, the pathogenic SNPs in PKCε were also predicted through bioinformatics tools that indicated its missence SNPs pathogenicity and potential impact on the structure and function of PKCε protein and non-coding variants impact on the gene expression of the protein [21, 22].

HCC is the most frequently occurring type of liver cancer accounting for ~ 85% of primary liver cancers. The risk factors associated with it include environmental exposures and hepatitis B and C viruses [23, 24]. Nearly 25% of HCC cases worldwide can be attributed to chronic HCV infection in patients. Chronic HCV infection results in development of inflammation-induced lesions in the liver which causes steatohepatitis leading to fibrosis. Over the span of many years, these events progress to cirrhosis or HCC in some patients [25, 26]. Studies at molecular levels have shown the involvement of PKCs in the progression of HCC [27], and research demonstrates that PKCε is engaged in various mechanisms leading to the progression of HCC [28, 29]. However, there are no known studies investigating the association of PRKCE SNPs with risk of HCC.

Based on evidence that nsSNPs in other PKC isoforms are correlated with the risk of cancers, we examined the association of a non-synonymous SNP (snSNP) in PRKCE, i.e. rs1553369874, in HCV-induced HCC in Pakistani population. Previously, nsSNP rs1553369874 was predicted as deleterious through several bioinformatics tools [21]. The study also indicated this SNP impact on the PKCε stability, flexibility, structure, and function. Therefore, this nsSNP was selected to probe its association with HCV-induced HCC.

## Methods

### Sample collection

Project approval was received from the Institution Review Board (IRB) of ASAB, NUST. Only patients with HCV-induced HCC were included in this research and written consent was taken from them prior to sample acquisition. HCV negative HCC cases were ignored as they were not the part of the hypothesis of this study. The control group taken was of healthy people without any known serious health or liver condition. Furthermore, a total of 63 patients belong to stage 1&2 and 37 from stage 3&4. None of the patients received disease treatment. Blood samples were acquired from patients of both genders whose ages were between 20 and 60 years. One hundred samples from HCC patients and an equal number of healthy control samples were collected for carrying out the procedures for identification and association of selected PKCε SNP with HCV-induced HCC.

### DNA extraction

Genomic DNA was extracted from blood using a phenol-chloroform extraction method [30]. This two day procedure utilizes four types of buffer solutions (namely A,B,C, and D), SDS, and proteinase K. Solution A was composed of 0.32 M sucrose, 5mM magnesium chloride, Triton X-100 and Tris (pH 7.5). Solution B consisted of 400mM sodium chloride, 2mM EDTA, and Tris (pH 7.5) while solution C solely consisted of phenol. Solution D was a mixture of isoamylalcohol and chloroform. 750 µl of blood was processed with solution A, B, 20% sodium dodecyl sulfate (SDS) and proteinase K on the first day followed by overnight incubation at 37 °C. On the second day the processing was completed using solution C, D, and DNA pellet was precipitated, washed, and dried using sodium acetate, chilled iso-propanol and ethanol in multiple steps. DNA was stored at 4 °C. The presence of DNA was confirmed with gel electrophoresis through 1% agarose at 120 volts. The quantification of DNA was performed on NanoDrop™ 8000 Spectrophotometer (Thermofisher scientific). Nanodrop value near 1.8 for 280/260 ratio was considered pure for extracted genomic content.

### Genotype analysis

For the analysis of PKCε gene using tetra ARMS-PCR, four primers were designed using primer 1 software. Forward inner (A allele) CTTCTTAAGATCAAAATCTTCA, forward outer CACAAGGTGTAGGGAGTGT, reverse inner (G allele) CTTCAAGCTCACGGCATC, and reverse outer GCTGTTGGTCTTCTGCTT. The tetra ARMS PCR reaction mixture contained 2 µl PCR buffer, 4.5 µl MgCl_2_, 2 µl dNTPs, 0.4 µl Taq Polymerase, 1 µl of each of the four primers, 1.5 µl the DNA sample and 10.6 µl PCR water. The PCR reaction entailed initial denaturation for 5 min at 92 °C, 35 cycles for 30 s each with denaturation 95 °C, annealing 50 °C, and extension at 72 °C along with the final extension at 72 °C for 7 min. PCR products were visualized by 2% (w/v) agarose gel electrophoresis.

### Measurement of ALT levels

Alanine aminotransferase (ALT) levels of both control and disease samples were measured to detect the change in liver functions of the HCC patients. An ALT assay kit by Merck (Darmstadt,

Germany) was used for this purpose. Blood serum from each of the samples was taken for ALT measurement. The chemical reaction between serum and kit reagents produced lactate as the end product and its absorbance was measured at 340 nm using a (UV-visible) spectrophotometer (Microlab 300 – Semi-automated chemistry analyzer). The concentration of ALT was calculated according to the formula, where kit factor is 1746 for any absorbance using 340 nm wavelength at room temperature:

$$\mathbf{c}\mathbf{o}\mathbf{n}\mathbf{c}\mathbf{e}\mathbf{n}\mathbf{t}\mathbf{r}\mathbf{a}\mathbf{t}\mathbf{i}\mathbf{o}\mathbf{n}= \varDelta \mathbf{A}\mathbf{b}\mathbf{s}/\mathbf{m}\mathbf{i}\mathbf{n}\mathbf{*}\mathbf{k}\mathbf{i}\mathbf{t} \mathbf{f}\mathbf{a}\mathbf{c}\mathbf{t}\mathbf{o}\mathbf{r}$$

Normal ALT values lie below 40–41 IUL^− 1^.

### Viral RNA extraction

The viral load was measured in HCV-induced HCC blood samples for comparison and association of SNPs with the severity of disease. For that, RNA of hepatitis C virus was extracted from the blood serum of the patients using Favorgen kit. The extracted RNA was dissolved in RNase free water and stored at -20 °C for qRT-PCR.

### Quantitative reverse transcriptase polymerase chain reaction (qRT-PCR)

Extraction of viral genome was performed from the blood plasma of patients through Instant virus RNA kit CE IVD ªAnalytik Jena and qRT-PCR employing TaqMan probes (TaqMan™ Fast Virus 1-Step Master Mix) was used for quantifying the viral RNA in real time. PCR reaction mix concentration and protocol for reaction was used as per the manufacturer’s instructions. Two standards with known concentrations were used to determine the upper and lower limit of the concentrations obtained from the samples. The CT (cyclic-threshold) value was determined for the samples; those samples with lower CT value contain more viral load than samples with higher CT value.

### Statistical analysis

GraphPad prism 9.0 (GraphPad, California, USA) and Microsoft Excel (Microsoft cooperation 2016) were employed for statistical analysis and making graphs. Frequency distribution of genotypes, odds ratio, relative risk along with the genotypes association with the disease was found out through Fisher Exact test. P-values below 0.05 were taken as significant.

## Results

### Genotype analysis

The data from tetra ARMS PCR was collected and statistical tools were applied for exploring its association with HCV induced HCC, which is presented in Tables 1 and 2. The comparitive genotype analysis between the control and patient sample data of all alleles of the selected locus of PKCε gene showed significance (P < 0.05) of the acquied data for all 3 genotypes of that locus. The homozygous wild genotype GG demonstrated high association with HCC with an odds ratio 4.19 and relative risk 2.1 (P < 0.0001). The other two genotypes were calculated to have odds ratio and relative risk less than 1, suggesting they are not associated with HCC development and progression (Table 1).

**Table 1:** Statistical analysis of genotyping data between HCC patients and control

Total Patient and Control Data							
**Genotype**	**Frequency Distribution %**		**Odds Ratio**		**Relative Risk**		**P value**
	**Patients**	**Control**	**Value**	**95% CI**	**Value**	**95% CI**	
**AA**	16.16%	32.32%	0.4036	0.2100-0.8024	0.6024	0.3837–0.8877	0.0124
**GG**	72.00%	38.00%	4.195	2.264–7.468	2.104	1.527–2.981	< 0.0001
**AG**	12.00%	30.00%	0.3182	0.1535–0.6541	0.5130	0.3031–0.8039	0.0029

**Table 2:** Statistical analysis of genotyping of PRKCE SNP with respect to gender

Patient and Control Data with respect to Gender							
**Genotype**	**Frequency Distribution %**		**Odds Ratio**		**Relative Risk**		**P value**
	**Patients**	**Control**	**Value**	**95% CI**	**Value**	**95% CI**	
**AA (F)**	20.00%	30.19%	0.5781	0.2237–1.472	0.7404	0.4168–1.188	0.2635
**GG (F)**	64.00%	38.89%	2.794	1.262–6.054	1.711	1.130–2.675	0.0118
**AG (F)**	16.00%	31.48%	0.4146	0.1607–1.106	0.6019	0.3144–1.028	0.0716
**AA (M)**	12.24%	34.78%	0.2616	0.09103–0.7278	0.4630	0.2186–0.8535	0.0141
**GG (M)**	80.00%	36.96%	6.824	2.766–15.84	2.737	1.642–4.919	< 0.0001
**AG (M)**	8.00%	28.26%	0.2207	0.07444–0.6796	0.4041	0.1615–0.8405	0.0146

Gender based comparison of the genotype data of control and patient samples demonstrated significant difference in the data of all three genotypes in males and GG genotype in females (P < 0.05). Concerning the GG genotype, the data depicted a slight difference in males and females, with the odds ratio and relative risk of GG association with HCC higher in males than in females. In males the odds ratio of GG is 6.824 and relative risk is 2.737, whereas in females the odds ratio is 2.794 and relative risk is 1.711. The P-value of other two genotypes AG in both males and females, and AA in males show their protective significance in HCV-induced HCC (Table 2). The results of genotype data obtained from tetra ARMS PCR was also analyzed with respect to the stages of HCC. The result of this analysis shows significance of the data for all genotypes in all stages of HCC except for genotype AA and AG in stages 3 and 4. The data shows that there is a high odds ratio and relative risk of GG genotype in all 4 stages of HCC (Table 3).

**Table 3:** Association of PRKCE variant rs1553369874 with HCV-induced HCC stages

Genotype	Frequency Distribution %		Odds Ratio		Relative Risk		P value
	Patients	Control	Value	95% CI	Value	95% CI	
**AA (stage 1&2)**	14.29%	32.32%	0.3490	0.1476 to 0.7616	0.4919	0.2615 to 0.8597	0.0101
**GG (stage 1&2)**	76.19%	38.00%	5.221	2.538 to 10.70	2.865	1.793 to 4.733	< 0.0001
**AG (stage 1&2)**	9.52%	30.00%	0.2456	0.09984 to 0.6320	0.3713	0.1717 to 0.7348	0.0019
**AA (stage 3&4)**	18.92%	32.32%	0.4885	0.1934 to 1.169	0.5803	0.2751 to 1.146	0.1410
**GG (stage 3&4)**	64.86%	38.00%	3.012	1.396 to 6.627	2.233	1.263 to 4.013	0.0067
**AG (stage 3&4)**	16.22%	30.00%	0.4516	0.1752 to 1.152	0.5430	0.2444 to 1.119	0.1280

### ALT levels analysis

**Fig. 1:**
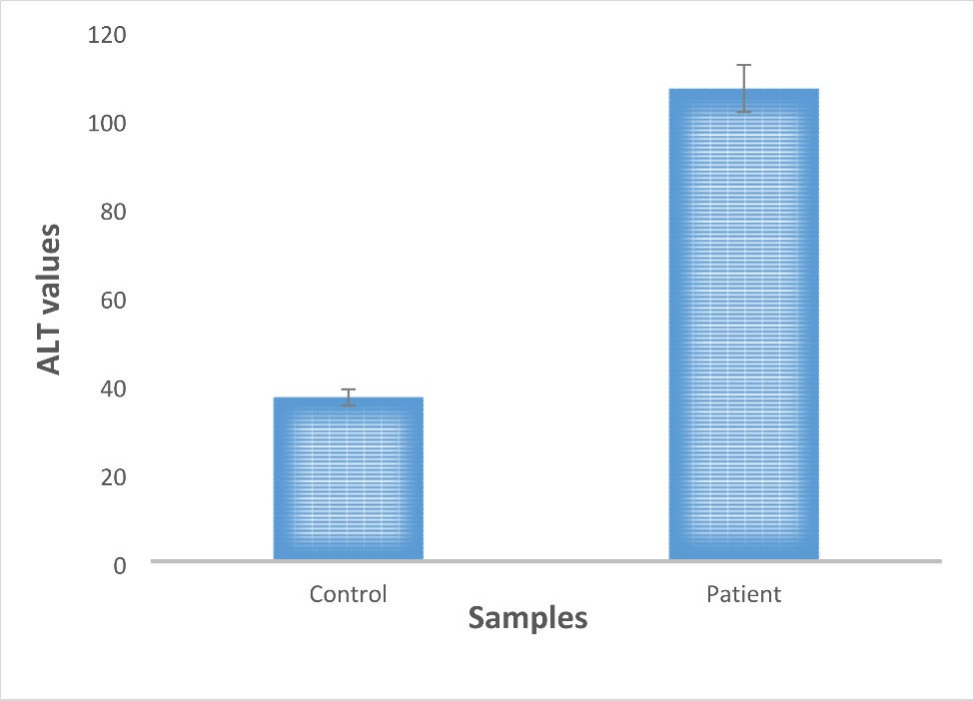
Graphical representation of a comparison between the ALT levels in HCV induced HCC patients versus control samples. ALT levels were found to be higher in patients with HCC.

ALT level test is an auxillary test that is performed to check the deviation in the normal liver function. It was performed for only functional analysis of the liver and has no correlation with the PKCε gene expression. The mean levels of ALT in control versus HCV-induced HCC patients is illustrated in Fig. 1. The ALT values of the control group (37.5 ± 10.2) compared to those of HCV-induced HCC patients (106.84 ± 52.5) indicate a considerable difference between the groups.

### Viral load analysis

**Fig. 2:**
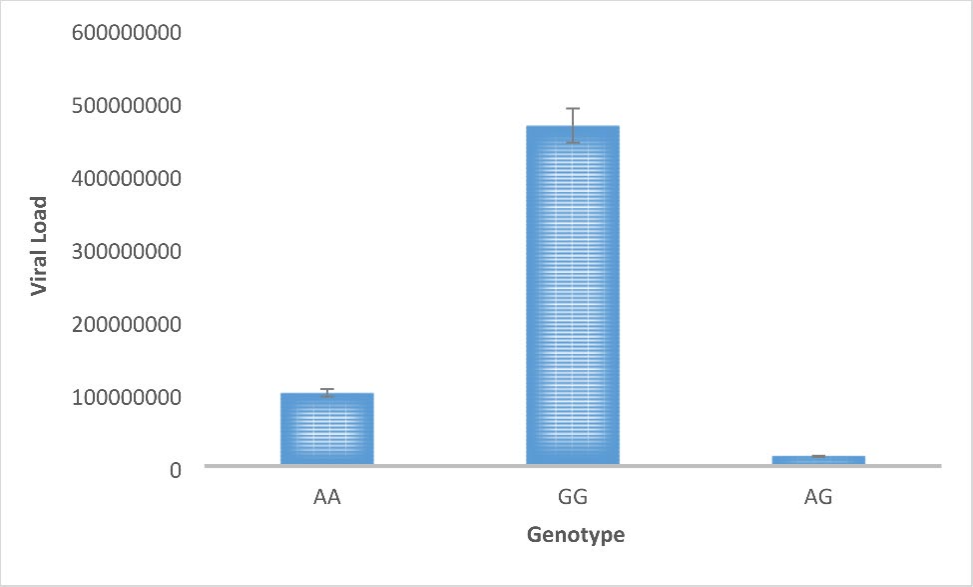
Viral load in patients with HCV induced HCC. Viral load is plotted against the three genotype variants of SNP rs1553369874. The graph shows that genotype GG is most prevalent in patients with HCV-induced HCC.

The viral load measured by qRT-PCR was plotted in the graph with respect to the three genotypes of the selected locus of PKCε as shown in Fig. 2. This graph shows clear association of the homozygous wild GG genotype with high abundance of viral load in HCV patients.

## Discussion

The presence of high-risk SNPs in PKCε represent a potential cause for its oncogenic properties observed in several types of cancers. From the mechanistic point of view, the role of PKCε in endothelial to mesenchymal transformation (EMT) may contribute to the invasive, migratory and metastatic properties of the cancer cells [8]. Its progressively elevated levels have been observed in the hyperplastic cells of rat liver in comparison to the normal liver cells which might have been associated with their malignant development [31]. However, the underlying cause can be a genetic polymorphism such as that present in several other genes which have been proved to be associated with development of disease and its progression. The presence of two novel SNPs in PKCε has been previously observed to be associated with the risk of occurrence of prostate cancer in the Han population [32]. Similarly, SNPs in five different genes involved in nucleotide excision repair pathways has been associated with the risk of development of HCC [33]. Therefore, we investigated a potentially damaging missense SNP of PKCε and examined the frequency of variants in HCV-induced HCC samples versus control samples.

Previously, a thorough *in silico* study to analyze the genetic variations of PKCε and their effects on protein structure, folding and function was carried out. Numerous *in silico* tools and software were employed for this purpose. The study aimed to identify potential disease-causing variants of PKCε gene for which the damaging non-synonymous variants were focused. The structure prediction of the protein with those variants along with further analysis exhibited the impact of variation on the dynamic behavior of protein. The delineation of phenotypic impact on PKCε due to the presence of variations helped in selecting a high-risk SNP that can further impact the functional properties of the protein [21].

The high-risk SNP was selected after screening through well-known and frequently used tools for this purpose [34, 35]. After careful screening the potential high-risk SNP of PKCε rs1553369874 was chosen for exploration of its association with HCV-induced HCC.

To see the damage inflicted on the liver of patients in comparison to the healthy individual, the ALT level of patient and control samples were measured. The results of this test provided a clear indication about the extent of liver damage in patients. Previous evidence suggests that ALT levels increase in patients with chronic liver damage and are significantly associated with the chronic HCV infection leading to HCC development [36].

Apart from that, elevated viral load with the GG genotype in HCV-induced HCC patients was found. This could be an indication of the increased susceptibility of idividuals with this genotype to hepatitis C viral pathogenicity. Previously, SNPs have been associated with increased viral load and disease progression in individuals with chronic viral infections [37, 38].

The statistical analysis of data obtained from ARMS-PCR after amplification of the selected SNP (rs1553369874) in 100 controls and 100 HCV-induced HCC patient’s samples pointed out that the homozygous wild genotype GG has significant association with HCV-induced HCC with 2.1 relative risk and 4.19 odds ratio. The analysis of this data with respect to gender also indicated the association of the same genotype in both males and females, although its relative risk in males and odds ratio is slightly greater than in females. This difference could also be due to the relatively greater incidence rate of liver cancer, more specifically HCC in males than in females. The male to female ratio of incidence and mortality of HCC exceeds 2.5 for both [39, 40].

## Conclusion

The damage sustained by the livers of HCV-induced HCC patients was confirmed though the measurement of ALT levels in them. The results from tetra ARMS PCR suggest the wild-type GG variant to be significantly associated with the disease progression. Viral load of this GG genotype was also very high among all three genotypes of HCV-induced HCC patients, indicating the increased susceptibility to disease in patients with this genotype. Thus, this SNP can be further explored for a potential biomarker for HCC prognosis by in vitro and in vivo studies. Investigation of a larger sample size along with the transcriptome and expression analysis of PKCε containing this SNP will provide better insight into its role in HCV-induced HCC occurrence.

## Data Availability

All data generated or analyzed during this study are included in this article.
